# Regulation of microRNA in systemic lupus erythematosus: the role of miR-21 and miR-210

**DOI:** 10.31138/mjr.31.1.71

**Published:** 2020-03-31

**Authors:** Maria Kourti, Maria Sokratous, Christina G. Katsiari

**Affiliations:** Department of Rheumatology and Clinical Immunology, Faculty of Medicine, University of Thessaly, Larissa, Greece

**Keywords:** microRNA, systemic lupus erythematosus, miR-21, miR-210, hypoxia, HIF-1a

## Abstract

miRNAs are small non-coding RNA molecules that participate through silencing in post-transcriptional regulation of gene expression. Recent studies have highlighted the importance of microRNAs (miRNAs) as regulators of both the innate and the adaptive immune response. There are emerging data regarding the role of miRNAs in patients with Systemic Lupus Erythematosus (SLE). One of the main stimuli for the induction of miR-21 is hypoxia. Moreover, the expression and function of miR-210 is directly related to the activity of “hypoxia inducible factor-1a” (HIF-1a). The aim of the study is to examine the regulation of miR-21 and mir-210 in patients with SLE based on the hypothesis that cellular hypoxia may have an important role in SLE pathogenesis. Plasma, PBMC and urine samples will be collected from patients with SLE and normal controls. miR expression will be studied with real-time PCR. Functional experiments will examine the effect of miR-21 and miR- 210 on HIFa and ERK1/2 και PI3K/AKT signalling pathways. The study will provide novel data regarding the expression and the role of miR-21 and miR-210 in patients with SLE. The results of the study will contribute to a better understanding of miR network regulation in SLE in order to ultimately identify molecules that can be used in clinical practice as diagnostic or prognostic markers, treatment response markers, or even as potential future therapeutic targets.

## BACKGROUND

Systemic lupus erythematosus (SLE) represents the prototype autoimmune disease, which can potentially harm every organ. Despite systematic research regarding pathophysiologic mechanisms in patients with SLE, the disease pathogenesis remains elusive.^1,2^ Recent studies have highlighted the importance of microRNAs (miRNAs) as regulators of both the innate and the adaptive immune response.^3^ miRNAs are small (21–25 nucleotides) non-coding RNA molecules that participate through silencing in post-transcriptional regulation of gene expression.^4^ miRNAs are stable molecules that can be detected in body fluids^5^ as well as in peripheral blood mononuclear cells (PBMC).^6^ The development of techniques for the detection and quantification of miRNAs has ignited great interest regarding the possible use of different miRNAs as biomarkers for early diagnosis of chronic diseases as well as biomarkers for the prognosis and response to treatment, with over 500 studies in progress (https://clinicaltrials.gov/ct2/results?term=miR&Search=Search).

Recent studies have examined the pattern of change of different miRNAs in PBMC, T cells, renal biopsies and plasma from patients with SLE.^7^ It is interesting to note that although dysregulation of several miRNAs has been found, the pattern of change differs among different studies. These differences could be attributed to several factors such as study population, disease state, type of biological sample as wells as technical pitfalls.^3^

Most studies in SLE have focused on the regulation of miRNAs in T cells regarding 4 main characteristics 3,8–10: 1) the induction of interferon pathway, implicating mir-146a, 2) DNA hypomethylation linked with increased expression of miR-21 and miR-148a in CD4+ T cells, 3) the impaired kinetics of CD40L surface expression related to increased miR-21^11,12^ and 4) IL-2 deficiency associated with overexpression of PP2Ac due to decreased expression of miR-155.^13,14^

## OBJECTIVE

The present study aims to examine the role of miR-21 and miR-210 in patients with SLE. One of the main stimuli for the induction of miR-21 is hypoxia.^15^ Moreover, hypoxia is characterized by the induction and function of hypoxia inducible factor-1a (HIF-1a). HIF-1a is necessary for the adaptation of cells to hypoxia in both normal and pathological conditions.^16,17^ The role of HIF-1a in SLE and possible correlation to miR-21 are unknown. Of note, HIF-1a was only recently found at high levels in the urine of patients with lupus nephritis.^18^ HIF-α activity is directly regulated by the ERK1/2 and PI3K/AKT kinases. In SLE, ERK1/2 kinase signalling pathway is dysfunctional with potential involvement in disease pathogenesis.^19,20^ Patients with SLE also display disequilibrium between Th-17 and T regulatory (Treg) cells.^21^ miR-21 regulates the differentiation of Th-17 cells, while HIF-1a has been proposed as an important regulator for the balance between Th17 and Treg cells.^22^

The expression and function of miR-210 is directly related to the activity of HIF-1a.^23^ Patients with psoriasis display increased levels of miR-210 in CD4 T cells, with FOXp3 as a gene target. Very little is known about miR-210 in SLE, such as the decreased expression in patients with nephritis.^24^ The present study will examine the levels of miR-21 and miR210 in plasma, urine and T cells from patients with SLE. Measurements will take place at 2 time points in order to examine possible correlations with disease activity, specific disease manifestations, and treatment. The role of miR-21 and miR-210 in the dysregulation of HIF-1a, ERK1/2 and PI3K/AKT kinases expression as well as TH17/Treg balance will be examined through silencing and overexpression experiments.

## METHODOLOGY

### Patients and Controls

Patients with SLE, according to the ACR classification criteria^25^ with disease duration for at least 6 months (n=50), and normal controls (n=30) will be included in the study. Any subject with any symptom or sign of infection or other autoimmune or inflammatory condition will be excluded. Blood samples will be drawn and urine samples will be collected, 24 hours following any medication. Samples from every patient will be collected at 2 different time points (interval > 3 months). Clinical (specific disease manifestations) and immunologic (autoantibody profile, complement level) parameters will be recorded and disease indices for activity (SLEDAI-2K, PGA) and damage (SLICC/ACR damage index) will be assessed.^26^

### Cell isolation and cultures

PBMC will be isolated over lymphoprep and T cells will be isolated using magnetic selection (Miltenyi Biotec). Cells will be cultured with stimuli such as PMA/ionomycin and anti-CD3/CD28 (Invitrogen). Cells will be transfected in order to silence or overexpress miR-21 or mir-210 (see paragraph “Transfection experiments”).

### Real time PCR

Real time rt-PCR will be used to quantify miR-21 and miR-210 in PBMC, T cells, plasma and urine (Qiagen).

### Flow cytometry

Flow cytometry (FACSCalibur, Becton Dickinson Mountain View, CA) will be used to examine cell pheno-type and in particular the balance between Th17/Treg cells. Intracellular Phosphospecific Flow Cytometry will be used to measure the phosphorylation of kinases ERK1/2 and Akt as well as the phosphorylation of transcription factor HIF-1a. Intact or permeabilized cells will be incubated with the appropriate fluorochrome-conjugated antibodies or their isotype controls (Molecular Probes, Invitrogen and BD Pharmingen). At least 0.5×105 cells will be collected from every sample for each measurement and BD CellQuest software (BD Bioscience) will be used for data acquisition and off-line analysis.

### Western blot

Western blotting will be used to measure protein levels of phosphorylated ERK1/2, AKT and HIF-1a as well as total amounts (including non-phosphorylated) of ERK1/2, Akt and HIF-1a employing appropriate moAbs (Invitrogen, Abcam).

### Transfection experiments

Transfection experiments: different transfection techniques will be tested to examine which is more efficient. Lipofection (Lipofectamine 2000, Thermo Fisher Scientific), a non-lipid polymer transfection method (JetPrime, Polyplus-transfection® SA) or electroporation will be tested. Silencing of miRNAs will be accomplished by synthetic monoclonal nucleic acids that specifically bind and inhibit endogenous miRNA molecules (Ambion® Anti-miR ™ miRNA, Thermo Fisher Scientific). Overexpression of miRNAs will be imposed via small, double-stranded RNA molecules (miRNAmimics, Qiagen)

### Statistical analysis

Statistical analysis will be performed using SPSS and GraphPad Prism Software.

## SIGNIFICANCE – PRELIMINARY AND ANTICIPATED RESULTS

The innovation of the research proposal includes the following

- The prospective nature of the study: measurements of miR-21 and miR-210 at different time points reflecting different disease activity. Such data are not available to date and may yield more reliable results on the ability of these miRs to be used as markers of activity and tools in patients’ follow-up.
- The study of a novel pathway of miR deregulation based on the hypothesis that cellular hypoxia may have an important role in SLE pathogenesis
- The study of miR-210 will generate novel data since very little is currently known

**Figure 1.**
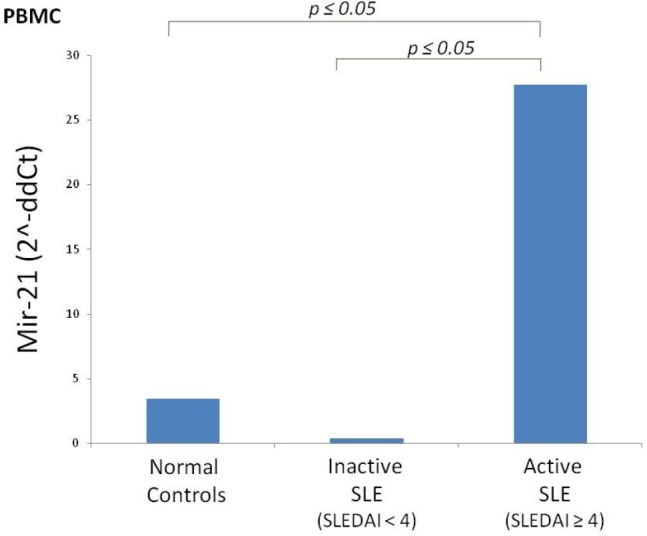
Increased expression of miR-21p in PBMC from patients with active SLE. Total RNA was extracted from PBMC derived from normal controls (=15) and patients with inactive (=24) and active (=14) SLE and expression of miR-21p was determined with real time PCR. SLEDAI-2k was used to determine the activity of the disease. Significant differences (p<0.05) are noted.

Preliminary results from the study of miR-21 have been analysed from data derived from 38 patients with SLE and 15 normal subjects. Experimental procedures were refined where, in brief, total RNA extraction from PBMCs (10^6^ cells) was done using NucleospinmiRNA kit (Macherey-Nagel, Duren, Deutschland) and from plasma samples (200μL) was performed using miRNeasy serum/plasma kit (Qiagen, Valencia, CA, USA) according to the manufacturer’s protocol. Before starting the miRNA extraction procedure, after addition of the denaturating solution, 3,5μL of the exogenous miRNA spiked-incontrol (cel-miR-39) was added to all the plasma aliquots to allow the normalization of sample-to-sample variation in the RNA isolation procedure. The quality and the concentration of extracted RNA was determined using nanodrop (Thermo scientific, USA). For mir-21 -5p detection, cDNA synthesis from total RNA was performed using Hispec buffer from miScript RT kit (Qiagen, Valencia, CA, USA) to obtain the cDNA from mature miRNA, according to the manufacturer’s instructions. Mir-21-5p expression levels were detected using miScriptSYBR Green PCR kit and miScript primer assays (Qiagen, Valencia, CA, USA). U6 was used as an internal control for normalization data from PBMCs samples. Mir-39 (Qiagen, Valencia, CA, USA) was used as external control for normalization data from plasma samples. All the PCR reactions were in a reaction volume of 20 μL and repeated two times. The relative expression levels of miR-21 were calculated using 2^−Δ ΔCt^ method. Patients with inactive disease display similar levels of miR-21, compared to healthy individuals both plasma and PBMC. On the contrary, patients with active disease display significantly higher levels of miR-21 in PBMC compared to both normal subjects and patients with inactive disease (*Figure 1*). The highest levels were detected in patients with very active disease (SLEDAI>12) and especially those that were treatment naive. Expression of miR-21 was also increased in the plasma collected from patients with active disease but the results were not significantly different when compared to normal subjects and inactive patients.

Overall, the proposed study will extend data regarding the expression of miR-21 in various biological materials and will reveal new molecular pathways that may be affected by this disorder. Along with the theoretical background of the existence of cellular hypoxia in inflammatory conditions, the study will produce novel data regarding the role of miR-210 specifically. The results of the study will contribute to a better understanding of miR network regulation in SLE in order to ultimately identify molecules that can be used in clinical practice as diagnostic or prognostic markers, treatment response markers, or even as potential future therapeutic targets.

## ABBREVIATIONS

HIF-1a: Hypoxia inducible factor-1a
miRNA: microRNA
PBMC: Peripheral blood mononu-clear cells
PGA: Physician’s Global Assessment
SLE: Systemic lupus erythematosus
SLEDAI: SLE Disease Activity Index
SLICC/ACR: Systemic Lupus Erythematosus International Collaborating Clinics/American College of Rheumatology
